# Mono- and Dinitro-BN-Naphthalenes: Formation and Characterization

**DOI:** 10.3390/molecules26144209

**Published:** 2021-07-11

**Authors:** Mao-Xi Zhang, Nathaniel B. Zuckerman, Philip F. Pagoria, Bradley A. Steele, I-Feng Kuo, Gregory H. Imler, Damon Parrish

**Affiliations:** 1 Lawrence Livermore National Laboratory, 7000 East Ave, Livermore, CA 94550, USA; zuckerman2@llnl.gov (N.B.Z.); pagoria1@llnl.gov (P.F.P.); steele26@llnl.gov (B.A.S.); kuo2@llnl.gov (I.-F.K.); 2 Naval Research Laboratory, 4555 Overlook Ave, Washington, DC 20375, USA; gregory.imler@nrl.navy.mil (G.H.I.); damon.parrish@nrl.navy.mil (D.P.)

**Keywords:** nitro-azaborine, nitro-BN-naphthalene, BN aromatics, nitration

## Abstract

Mono- and dinitro-BN-naphthalenes, i.e., 1-nitro-, 3-nitro-, 1,6-dinitro-, 3,6-dinitro-, and 1,8-dinitro-BNN, were generated in the nitration of 9,10-BN-naphthalene (BNN), a boron–nitrogen (BN) bond-embedded naphthalene, with AcONO_2_ and NO_2_BF_4_ in acetonitrile. The nitrated products were isolated and characterized by NMR, GC-MS, IR, and X-ray single crystallography. The effects of the nitration on the electron density and aromaticity of BNN were evaluated by B-11 NMR analysis and HOMA calculations.

## 1. Introduction

As part of a continuing program in the synthesis of nitro-substituted, boron–nitrogen (BN) bond-embedded aromatics as next-generation energetic materials, we undertook the nitration of 9,10-BN-naphthalene (BNN) with AcONO_2_ and NO_2_BF_4_ in acetonitrile. The nitration led to the formation of mono- and dinitro-BN-naphthalenes. To our knowledge, dinitro-BN aromatics have not been reported previously.

BN aromatics have been widely investigated in recent years since the materials possess attractive physical and chemical properties and hold potential applications in the light-emitting polymer [1,2,3,4,5,6,7,8,9,10,11,12], semiconductor [13,14,15,16,17], and other research fields [18,19,20,21,22,23,24,25,26,27,28,29]; however, there have been relatively few reports on the synthesis of nitrated BN aromatics. Thus far, only five mononitro-BN aromatics have been reported [30,31]. Dewar and their co-workers [30], in 1959, reported the first nitration of a BN aromatic, BN-phenanthrene (BNP), yielding a mixture of mononitro-substituted BNPs (**1**). In their work, the nitrating reagent, acetyl nitrate (AcONO_2_), was generated by treating Ac_2_O with HNO_3_ in acetic acid. This is a typical procedure for generating AcONO_2_ in organic synthesis, but in our hands, AcONO_2_ generated by this method failed to nitrate BN-naphthalene, giving only decomposition. Fang and co-workers [31], in 2017, successfully nitrated azaborazines to nitro-azaborazines (**2**) and BNN to 3-nitro-BNN (**3**) using AcONO_2_ generated from hydrated metal nitrates and acetyl chloride (AcCl) in CH_2_Cl_2_. The nitration provided highly positional selectivity at the *para*-position relative to the boron atom, but the nitration did not produce higher nitrated BN derivatives even at the nitration temperature of 95 °C [31].

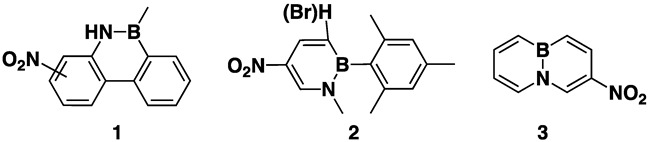



## 2. Results and Discussions

### 2.1. Nitration of BN-Naphthalene

As mentioned above, a successful nitration of boron–nitrogen bond-embedded aromatics critically depends on the nitration conditions. In our preliminary trial, it was found that BN-naphthalene is sensitive to certain nitration reagents, such as HNO_3_/H_2_SO_4_, N_2_O_4_/AcOH, HNO_3_/Ac_2_O/AcOH/, and HNO_3_/Ac_2_O. These acidic nitration conditions seem to decompose BNN. Therefore, neutral nitration conditions such as AcONO_2_ and NO_2_BF_4_ in acetonitrile were investigated in the hope that BNN would survive under the reaction conditions and produce higher nitrated BNN products.

Indeed, when AcONO_2_ was generated from AgNO_3_ and AcCl and the nitration was carried out in anhydrous acetonitrile, mono- and dinitro-BN-naphthalenes were formed. Using one equivalent of the nitration reagent, only mononitro-BNNs were observed, with about 50% conversion, as judged by GC-MS and NMR. By increasing the nitration reagent to 2.7 equivalents, the starting material BNN was consumed completely with the formation of a mixture of mono- and dinitro-BNNs. The procedure involved treating BNN [32] (1.7 mmol) with 2.7 equivalents of AcONO_2_ generated from 2.7 equivalents of AgNO_3_ and 3.1 equivalents of AcCl in anhydrous acetonitrile at 10 °C and stirring the mixture at 20 °C for 2 h. The products were isolated by silica gel column chromatography, eluting with CH_2_Cl_2_/hexane. The first product eluted was identified as the known 3-nitro-BNN (**3**) [31]. Further elution gave a second, previously unreported product, 1-nitro-BN-naphthalene (**4**). The molecular structure was confirmed by GC-MS and ^1^H and ^13^C NMR and further confirmed by X-ray crystallographic analysis (all spectra can be found in Supplementary Materials). The third product eluted from the column was assigned as 3,6-dinitro-BN-naphthalene (**5**) based on ^1^H and ^13^C NMR, and high-resolution mass spectroscopy (*m*/*z* = 219.0459, corresponding to dinitro-BNN: C_8_H_6_BN_3_O_4_, calculated 219.0451). Following compound **5**, two other dinitro-BNNs, 1,6- (**6**) and 1,8-dinitro-BNN (**7**), were isolated. Both structures were assigned by NMR, GC-MS, and X-ray single-crystal analysis. The ratio of **3**, **4**, **5**, **6**, and **7** isolated from the nitration is 45:23:6:16:11, with a total yield of 37%.

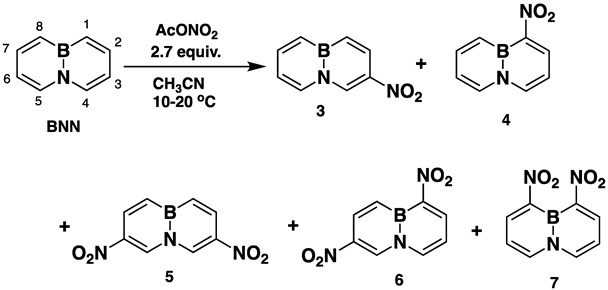



Further increasing the number of equivalents of the nitration reagent increases the amounts of dinitro-BNNs but decreases the overall yield. When the nitration reagent was increased to 6.0 equivalents, no detectable mono- and dinitro-BNNs nor trinitro- or higher nitrated BN derivatives were found.

Nitronium tetrafluoroborate (NO_2_BF_4_) is a relatively powerful nitration reagent which has been previously reported to give poly-nitro-substituted aromatics under mild conditions [33,34]. Applying the reagent to BNN and carrying out the nitration with three equivalents of NO_2_BF_4_ under similar reaction conditions to the AcONO_2_ nitration (anhydrous CH_3_CN, 10–20 °C), we isolated only 3,6-dinitro-(**5**) and 1,6-dinitro-BNN (**6**), with no detectable amounts of mononitro-BNNs nor 1,8-dinitro-BNN (**7**). The ratio of **5** and **6** is 53:47 based on ^1^H NMR integration, with a total isolated yield of 39%.

**Figure 1:**
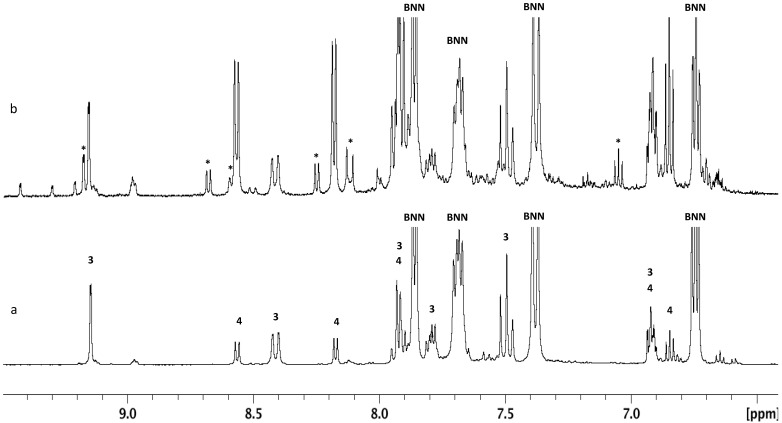
^1^ HNMR analysis of the nitration mixture from the reaction of BNN with AcONO_2_ and NO_2_BF_4_ in CH_3_CN at 10–20 °C. The products were measured in CD_2_Cl_2_. (**a**) AcONO_2_ prepared from 1.0 equiv. of AgNO_3_ and 1.1 equiv. of AcCl. (**b**) NO_2_BF_4_, 1.0 equivalent (dinitro-BNN (**6**) labeled as *).

To look more closely at the relative reactivities of the nitration reagents to BN-naphthalene, we carefully treated BNN with one equivalent of AcONO_2_ and NO_2_BF_4_, under identical reaction conditions, with the assumption that only mono-nitrated products would be formed. Indeed, AcONO_2_ nitration, according to NMR analysis, gave only mononitro-BNN (**3)** and (**4**) and un-reacted BNN. The ratio of **3**:**4**:BNN was estimated to be 34:17:49 by integration of the NMR signals (Figure 1a). Replacing AcONO_2_ with NO_2_BF_4_, we observed not only mono-nitrated products **3** and **4** but also dinitro-BNN (**6**), even though the starting material BNN was also not completely consumed. The product ratio of **3:4:6**:BNN was 23:33:13:31 (Figure 1b). Different from AcONO_2_ nitration, NO_2_BF_4_ nitration gave 1-nitro-BNN (**4**) as the major product, while AcONO_2_ nitration gave predominately 3-nitro-BNN (**3**).

### 2.2. The Spectral and Structural Features of Nitro-BNNs

The introduction of nitro groups to BNN results in a downfield chemical shift for all protons. Similar to carbon–carbon bond-based aromatics, nitro groups, being strongly electron-withdrawing, lower the electron density on the BNN heterocyclic rings, resulting in significant deshielding of the ring protons. However, the nitro groups do not move B-11 chemical shifts in the same manner; B-11 resonances are shifted both upfield and downfield depending on the position where the nitro group is attached. The chemical shift of BNN itself appears at 27.8 ppm (28.4 ppm in n-hexane and 27.9 ppm in AcOH [35]). The introduction of a nitro group to position-1 (**4**) results in a 2.2 ppm upfield shift to 25.6 ppm. A higher upfield shift of 5.2 ppm is observed when the second nitro group is introduced to position-8 (**7**), giving a B-11 chemical shift at 22.6 ppm. However, when the nitro group is attached to position-3 (**3**), the B-11 shifts downfield by 1.7 ppm to 29.5 ppm. Further introducing a nitro group to position-6 (**5**) leads to a further downfield shift by 2.1 ppm to 29.9 ppm (Table 1).

The chemical shift of B-11 in homogeneous solution has a close linear relationship with the boron π-electron density [36]. Applying Michl’s equation [37], the boron electron density for each nitro-BNN based on the chemical shifts was calculated (Table 1). This shows that the nitro groups attached to the carbons at position-1 and -8 increase the boron π-electron density, but at position-3 and -6, they decrease the density. Baranac-Stojanovic and Stojanovic [38] pointed out that nitro-1,2-azaborazine with a nitro group at the α-position to the boron atom is more stable than its isomer where the nitro group is in the para-position. It was reasoned that the electron-withdrawing nitro group on the carbon at the α-position to the boron atom results in the electron density being drawn to the electron-deficient boron from the neighboring nitrogen to provide a better electrostatic stabilization energy to the molecule [38].

The other important features of a BN aromatic compound are bond lengths and bond angles. These parameters are related to molecular aromaticity and stability. Therefore, we tried to prepare single crystals for all nitro-BNNs to determine the parameters, but only **4**, **6**, and **7** with a nitro group at position-1 provided crystals of sufficient quality for X-ray analysis. Crystals **4** and **6** are in the orthorhombic space group, with densities of 1.406 and 1.583 g/cm^3^, respectively. Compound **7** is monoclinic, with a density of 1.553 g/cm^3^ (BNN [39], *ρ* = 1.215 g/cm^3^). The crystallographic analysis confirms that the introduction of nitro groups to BNN rings does not significantly distort the structure of the molecule. The rings in **4** and **6** are completely planar. In **7,** the rings have a slight torsional angle of ~7.3° centered on the boron atom. The nitro groups on **4** and **6** are co-planar with BNN rings, but the nitro groups in **7** are rotated about 39° out of the plane of the heterocyclic rings due to the steric crowding of the nitro groups.

The bond lengths of **4**, **6**, and **7** are summarized in Table 2, along with the reported bond lengths of the parent BNN [39,40]. All other crystal structure data can be found in the supporting information.

**Figure 2:**
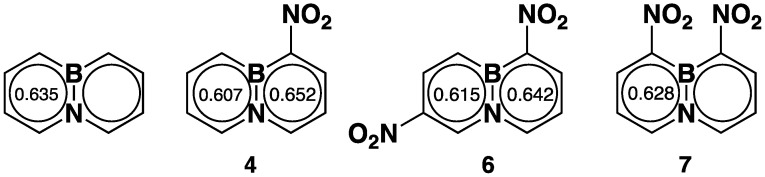
Aromaticity of nitro-BNNs calculated using HOMA model.

The B–N bond length is usually used as a measure of the relative stability of BN aromatics. Table 2 shows that the introduction of nitro groups to BNN does not significantly change the B–N bond length. When a nitro group is attached to BNN at position-1 (to **4**), the B–N bond length is lengthened slightly by 0.011 Å, from 1.461 to 1.472 Å. With a further introduction of a second nitro group to position-8 (to **7**), the length of the B–N bond is not lengthened but slightly shortened, changing from 1.472 to 1.460 Å. However, when the second nitro group is introduced to **4** at position-6 (to **6**), the B–N bond length falls between the B–N bond lengths of **4** and **7** at 1.469 Å (Table 1). To look closely at how the bond lengths of nitro-BNNs impact the molecular aromaticity, we introduced the HOMA index [41,42] by using bond lengths collected from the X-ray crystallographic analysis. The results are depictured in Figure 2.

According to HOMA calculations, BN-naphthalene (BNN) possesses 81% of the aromaticity of naphthalene [41]. The introduction of one nitro group to position-1 slightly changes the aromaticity of BNN (±2%). The same results were observed when the second nitro group was introduced to position-6 or -8, suggesting that dinitro-BNNs retained a significant amount of aromaticity.

## 3. Conclusions

We demonstrated the first reported formation of dinitro-substituted-BN-naphthalenes using AcONO_2_ and NO_2_BF_4_ as the nitrating reagents in acetonitrile. These new boron–nitrogen bond-embedded nitrocompounds were fully characterized by NMR, GC-MS, IR, and X-ray crystallographic analysis. In addition, the positional effect of nitro substitution on the electron density of the boron atom of BNN, along with a comparison of the positional effect of nitration on the aromaticity of nitrated BNN with respect to the parent BNN, was discussed.

## 4. Experimental Section

All reagents and anhydrous solvents were purchased from commercial suppliers and used without further purification except nitronium tetrafluoroborate (NO_2_BF_4_). The purity of acetyl chloride was ≥ 99%, AgNO_3_ ≥ 99%, and anhydrous CH_3_CN, ≥ 99.9%. NO_2_BF_4_ was purified by washing the commercial product with anhydrous nitromethane and CH_2_Cl_2_ under argon before use. NO_2_BF_4_ (commercial product, ≥ 95%, 5 g) was placed into a Schlenk tube equipped with a fritted filter. Anhydrous nitromethane (10 mL) was added, and the mixture was stirred with a glass bar under argon for 10–20 s. The liquid was then drawn under argon. The solid was washed with anhydrous nitromethane (10 mL) for one more time, followed by anhydrous CH_2_Cl_2_ (3 × 10 mL), and dried under slow-flowing argon for 2 h. 9,10-BN-Naphthalene was prepared from di-(3-butene-1-yl)amine using a slight modification of Dewar’s method [32] and purified by column chromatography (silica gel, CH_2_Cl_2_:Hexane, 1:5, R_f_ = 0.5), followed by recrystallization from cold pentane.

^1^H and ^13^C NMR spectra were acquired on either a Bruker 500 MHz spectrometer (500 and 150 MHz, respectively) or an Anasazi Instruments Eft-90 MHz spectrometer with a Varian magnet (90 and 22.5 MHz, respectively). ^11^B NMR was performed on an Anasazi Instruments Eft-90 MHz spectrometer (28.1 MHz) using BF_3_.Et_2_O as the reference (0 ppm). ^1^H and ^13^C NMR chemical shifts were reported relative to the residual solvent as internal standard, such as CD_2_Cl_2_ (5.32 ppm for proton and 53.5 ppm for C-13). Infrared spectra (thin films) were collected using a Bruker Alpha ZnSe ATR FTIR. GC-MS was performed on an Agilent 7890A gas chromatograph equipped with a 5975 mass spectrometer and an NCI detector.

X-ray crystallography: Colorless crystals were mounted on α MiteGen MicroMesh by using a small amount of Cargille Immersion oil. Data were collected on a Bruker three-circle platform diffractometer equipped with a SMART APEX II CCD detector. The crystals were irradiated by using graphite-monochromated MoKα radiation (λ = 0.71073). An Oxford Cobra low-temperature device was used to maintain the crystals at a constant 150(2) K during data collection. Data collection was performed, and the unit cell was initially refined by using APEX2 (v2010.3–0). Data reduction was performed using SAINT (v7.68 A) and XPREP (v2008/2). Corrections were applied for Lorentz, polarization, and absorption effects by using SADABS (v2008/1). The structure was solved and refined with the aid of the programs in the SHELXTL-plus (v2008/4) system of programs. The full-matrix least squares refinement on F2 included atomic coordinates and anisotropic thermal parameters for all non-hydrogen atoms.

### 4.1. Nitration of BNN with AcONO_2_

A flask equipped with a thermometer, gas inlet, and stir bar was charged with BN-naphthalene [5] (0.22g, 1.7 mmol), anhydrous CH_3_CN (7 mL), and AgNO_3_ (0.79 g, 4.7 mmol). With vigorous stirring, acetyl chloride (0.42 g, 5.4 mmol) in 1 mL of anhydrous CH_3_CN was added dropwise at 10 °C. The reaction temperature did not change during the addition. The nitration mixture was warmed to 20 °C and stirred for 2.0 h. The precipitated AgCl was removed by filtration through a layer of Celite and the solvent was removed in vacuo. The oily residue was diluted with CH_2_Cl_2_ (20 mL), quickly washed with water (2 × 3 ml), and dried over MgSO_4_. After filtration and concentration, the residue was loaded onto a silica gel column chromatograph and eluted with a 2:1 CH_2_Cl_2_ and hexane solvent mixture. The first fraction was identified as compound 3-Nitro-BN-naphthalene (**3**) [31] (R_f_ = 0.57), the second was 1-Nitro-BN-naphthalene (**4**) (R_f_ = 0.37), the third was 3,6-Dinitro-BN-naphthalene (**5**) (R_f_ = 0.26), and the fourth was 1,6-Dinitro-BN-naphthalene (**6**)(R_f_ = 0.17). Compound 1,8-Dinitro-BN-naphthalene (**7**) (R_f_ = 0.09) was eluted with CH_2_Cl_2_. The total yield was 37%.

*3-Nitro-BN-naphthalene* (**3**), white solid, 50.0 mg (17%), mp. 78–79 °C. ^1^H NMR (CD_2_Cl_2_): δ 9.14 (s, 1H), 8.41 (d, *J* = 12.5 Hz, 1H), 7.92 (d, *J* = 6.9 Hz, 1H), 7.79 (q, *J* = 11.0, 6.5 Hz, 1H), 7.49 (q, *J* = 12.3, 11.0 Hz, 2H), 6.92 (t, *J* = 6.7 Hz, 1H). ^13^C NMR (CD_2_Cl_2_): δ 140.75, 138.96 (C-NO_2_), 136.05, 134.13, 132.23, 131 (b, C-B), 117.08. ^11^B NMR (CD_2_Cl_2_) δ 29.52. GC-MS *m*/*z* (EI): 174.2 (m, 100%), 173.2 (22%). IR (film) 1627, 1537, 1515, 1473, 1399, 1336, 748 cm^−1^.

*1-Nitro-BN-naphthalene* (**4**), white solid, 25.0 mg (8%), mp. 124–126 °C. ^1^H NMR (CD_2_Cl_2_): δ 8.56 (d, *J* = 7.6 Hz, 1H), 8.18 (d, *J* = 6.8 Hz, 1H), 7.93 (d, *J* = 11.3 Hz, 1H), 7.91 (d, *J* = 7.1 Hz, 1H), 7.86 (q, *J* = 11.4, 6.3 Hz, 1H), 6.91 (t, *J* = 6.8, 1.3 Hz, 1H), 6.84 (t, *J* = 7.3 Hz, 1H). ^13^C NMR (CD_2_Cl_2_): δ 140.97, 140.92, 137.40, 133.59, 131.0 (b, C-B), 116.14, 110.71. ^11^B NMR (CD_2_Cl_2_) δ 25.63. GC-MS *m*/*z* (EI): 174.1 (m, 100%), 173.1 (27%). IR (film) 1617, 1502, 1333, 1313, 1232, 751cm^−1^.

*3,6-Dinitro-BN-naphthalene* (**5**), white solid, 8 mg (2%), mp. 191–192 °C. ^1^H NMR (CD_2_Cl_2_): δ 9.21 (s, *J* = 1.5 Hz, 2H), 8.51 (d, *J* = 12.5 Hz, 2H), 7.65 (d, *J* = 12.5 Hz, 2H). ^13^C NMR (CD_2_Cl_2_): δ 140.84 (C-NO_2_), 135.81, 134.50, 133 (b, C-B). ^11^B NMR (CD_2_Cl_2_) δ 29.91. GC-MS *m*/*z* (EI): 219.0 (m, 100%), 218.0 (23%). HRMS *m*/*z* = 219.0459, corresponding to dinitro-BNN: C_8_H_6_BN_3_O_4_, calculated 219.0451. IR (film) 1625, 1529, 1337, 1240, 1110, 835 cm^−1^.

*1,6-Dinitro-BN-naphthalene* (**6**), white solid, 22 mg (6%), mp. 179–180 °C. ^1^H NMR (CD_2_Cl_2_): δ 9.17 (s, 1H), 8.68 (d, *J* = 7.5 Hz, 1H), 8.58 (d, *J* = 12.5 Hz, 1H), 8.25 (d, *J* = 7.0 Hz, 1H), 8.12 (d, *J* = 12.5 Hz, 1H), 7.05 (dd, *J* = 7.0, 7.5 Hz, 1H). ^13^C NMR (CD_2_Cl_2_): δ 140.83, 140.11, 139.52, 135.09, 134.76, 132.0 (b, C-B), 113.63. ^11^B NMR (CD_2_Cl_2_) δ 26.64. GC-MS *m*/*z* (EI): 219.0 (m, 100%), 218.0 (29%). IR (film) 1625, 1529, 1337, 1240, 1110, 835, 800 cm^−1^.

*1,8-Dinitro-BN-naphthalene* (**7**), white solid, 15 mg (4%), mp. 250 °C (decomp). ^1^H NMR (CD_2_Cl_2_): δ 8.33 (d, *J* = 7.5 Hz, 2H), 8.13 (d, *J* = 7.0 Hz, 2H), 6.97 (dd, *J* = 7.0, 7.5 Hz, 2H). ^13^C NMR (CD_2_Cl_2_): δ 138.66, 137.64, 112.64. ^11^B NMR (CD_2_Cl_2_) δ 22.58. GC-MS *m*/*z* (EI): 219.0 (m, 100%), 218.0 (29%). IR (film) 1618, 1500, 1363, 809, 744, 693 cm^−1^.

### 4.2. Nitration of BNN with NO_2_BF_4_

A flask equipped with a gas inlet and a stir bar was charged with BN-naphthalene (9.0 mg, 0.07 mmol) and anhydrous CH_3_CN (0.5 mL). The mixture was stirred in a water bath (10 °C), and NO_2_BF_4_ (28 mg, 0.21 mmol) was added in one portion. The reaction mixture was warmed to 20 °C and stirred at the temperature for 2 h. The reaction mixture was concentrated in vacuo at 20 °C, and the residue was diluted with 1.0 mL of ice water. The products were extracted with methylene chloride, washed with water, and dried over MgSO4. The drying reagent was filtered, and the filtrated product was passed through a layer of silica gel. The solvent was removed in vacuo to give a solid, 6.0 mg. The NMR spectrum showed that the mixture only contained 3,6-dinitro- (**5**) and 1,6-dinitro-BNN (**6**). The total yield was about 39%.

### 4.3. Nitration of BNN with One Equivalent of AcONO_2_ in CH_3_CN

Under an argon atmosphere, AcCl (6.6 mg, 0.084 mmol) in 0.29 mL of anhydrous CH_3_CN was added dropwise via a syringe to a solution of BNN (10 mg, 0.078 mmol) and AgNO_3_ (13.1 mg, 0.078 mmol) in 0.2 mL of anhydrous CH_3_CN at 10 °C. After the addition was completed, the water bath was warmed to 20 °C, and the reaction mixture was stirred for 2.0 h. The solvent was removed in vacuo at 20 °C, and the residue was diluted with 2 mL of CH_2_Cl_2_ and washed with water (2 × 0.5 mL). The organic phase was dried over MgSO_4_. The drying reagent was filtered, and the filtrate was treated by passing through a layer of silica gel, eluted with CH_2_Cl_2_. Removal of the solvent gave an oily mixture, 13 mg. The mixture was dissolved in CD_2_Cl_2_ for NMR analysis (Figure 1a).

### 4.4. Nitration of BNN with One Equivalent of NO_2_BF_4_ in CH_3_CN

Under an argon atmosphere, NO_2_BF_4_-CH_3_CN solution, prepared from 100 mg of purified NO_2_BF_4_ in 2.73 g of anhydrous CH_3_CN (0.218g, 0.058 mmol of NO_2_BF_4_, assume the purity of NO_2_BF_4_ is 100%), was added dropwise from a syringe to a solution of BNN (7.0 mg, 0.054 mmol) in 0.2 mL of anhydrous CH_3_CN at 10 °C. After the addition was completed, the reaction mixture was warmed to 20 °C, and the reaction mixture was stirred for 2.0 h. The solvent was removed in vacuo at 20 °C. The residue was diluted with 2 mL of CH_2_Cl_2_ and washed with water (2 × 0.5 mL). The organic phase was dried over MgSO_4_, the drying reagent was filtered, and the filtrate was treated by passing through a layer of silica gel and eluted with CH_2_Cl_2_. Removal of the solvent gave an oily mixture, 8 mg. The mixture was dissolved in CD_2_Cl_2_ for NMR analysis (Figure 1b).

## Figures and Tables

**Table 1:** ^11^ B NMR chemical shifts of nitrated BNN and corresponding boron π-electron density.

BNNs	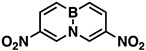	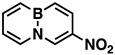	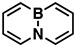	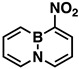	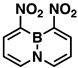
^11^B NMR (ppm)	29.9	29.5	27.8	25.6	22.6
Boron *q*	0.518	0.524	0.548	0.580	0.624

**Table 2:**
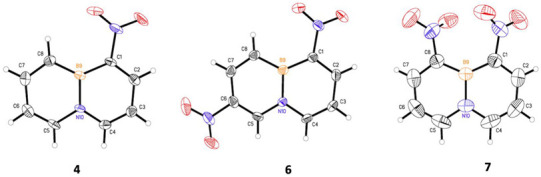
Bond lengths of **4**, **6**, and **7** from X-ray crystallographic analysis ^a^.

	Bond Type and Bond Length (Å) ^a^										
	BC_1_	C_1_C_2_	C_2_C_3_	C_3_C_4_	C_4_N	BN	C_5_N	C_5_C_6_	C_6_C_7_	C_7_C_8_	BC_8_
BNN ^b^ BNN ^c^	1.455 1.510	1.357 1.352	1.431 1.435	1.360 1.384	1.445 1.391	1.461 1.470	1.445 1.391	1.361 1.384	1.431 1.435	1.357 1.352	1.455 1.510
**4**	1.525	1.358	1.415	1.359	1.382	1.472	1.395	1.343	1.427	1.364	1.510
**6**	1.517	1.368	1.412	1.344	1.395	1.469	1.381	1.353	1.423	1.350	1.520
**7**	1.520	1.356	1.396	1.337	1.387	1.460	1.387	1.337	1.396	1.356	1.520

^a^ All X-ray crystallographic data can also be found at the Cambridge Crystallographic Data Center with the following codes: **4**, 1977407; **6**, 1977408; and **7**, 1977409. ^b^ Ref. [39], the data were from X-ray crystallographic analysis. Due to the disorder of the molecule, the boron–carbon and nitrogen–carbon bond lengths are averaged. ^c^ Ref [40], the data were obtained from a microwave measurement.

## Data Availability

Not applicable.

## Supplementary Materials

^1^H, ^13^C, and ^11^B NMR and IR spectroscopy of compounds **3**–**7**, and X-ray crystallographic analysis data (bond lengths and bond angles of **4**, **6**, and **7**) are available in the supporting information.

## Sample Availability

Samples of the compounds 3, 4, 5, 6, and 7 are not available from the authors.
