# Supplementary material for: Mono- and Dinitro-BN-Naphthalenes: Formation and Characterization
Source: Molecules. 2021 Jul 11;26(14):4209. doi: 10.3390/molecules26144209 (PMC8303104; doi:10.3390/molecules26144209)
Supplement: Supplementary file 1 [file molecules-26-04209-s001.zip › molecules-1280049-supplementary.pdf]

Supplimentary Materials

Mono- and Dinitro BN-Naphthalenes: Formation and Characterization

Mao-Xi Zhang, † * Nathaniel B. Zuckerman, † Philip. F. Pagoria, † Bradley A. Steele, † I-Feng Kuo, † Gregory H. Imler, ‡ and Damon Parrish ‡

†Lawrence Livermore National Laboratory, 7000 East Ave, Livermore, California 94550, United States.
‡ Naval Research Laboratory, 4555 Overlook Ave, Washington DC 20375, United States

Content

1. Proton NMR of nitro-BNNs
2. Carbon-13 NMR of nitro-BNNs
3. B-11 NMR of nitro-BNNs
4. Infrared spectra of nitro-BNNs
5. X-ray crystallography of BNNs

1. *Proton NMR spectra of compound **3**, **4**, **5**, **6**, and 7.*

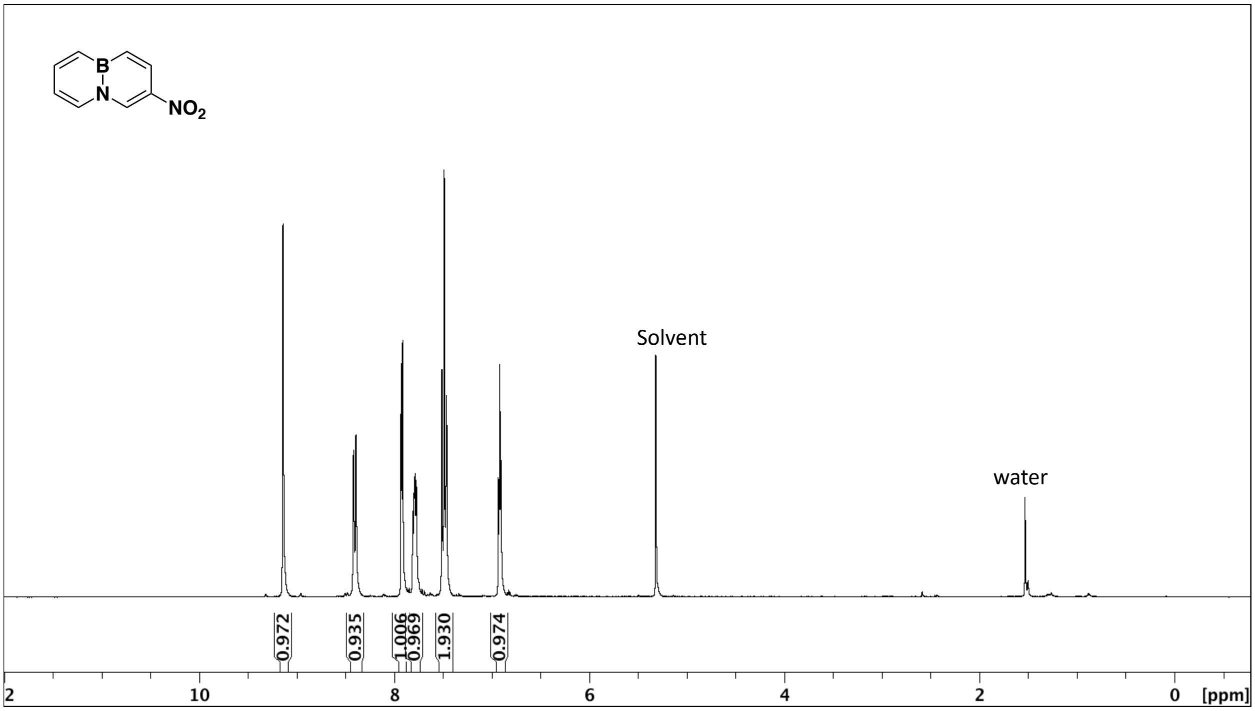

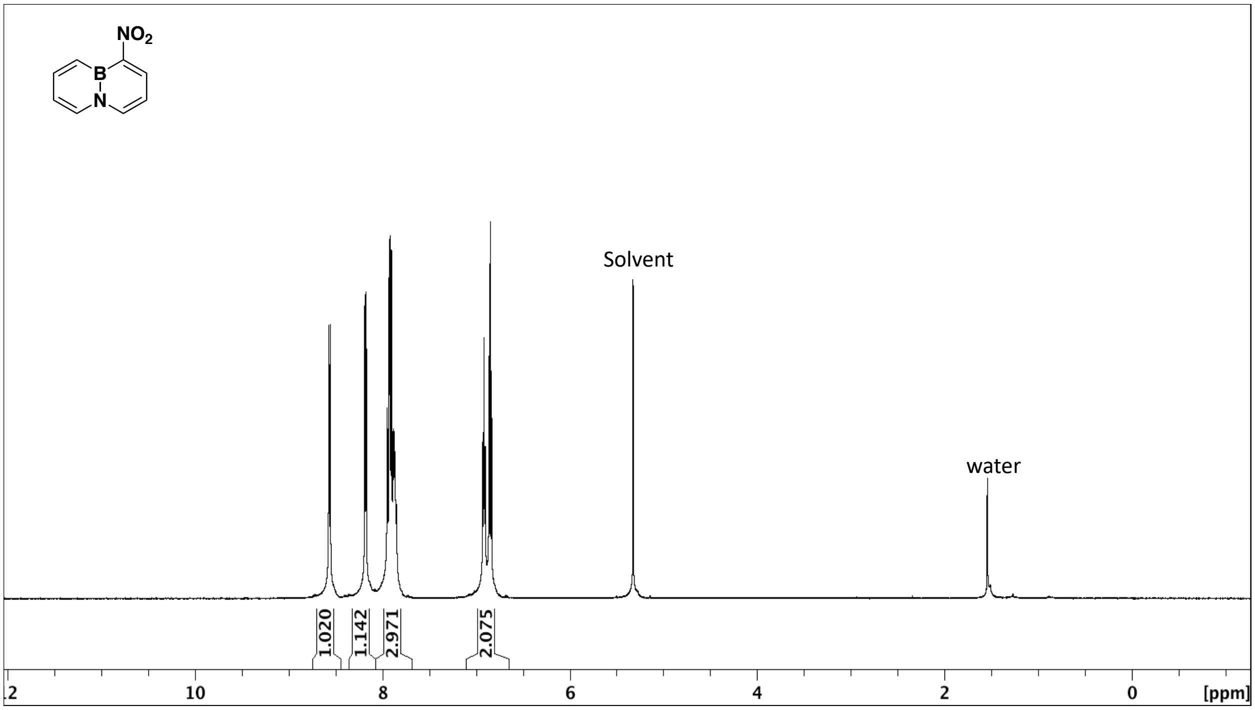

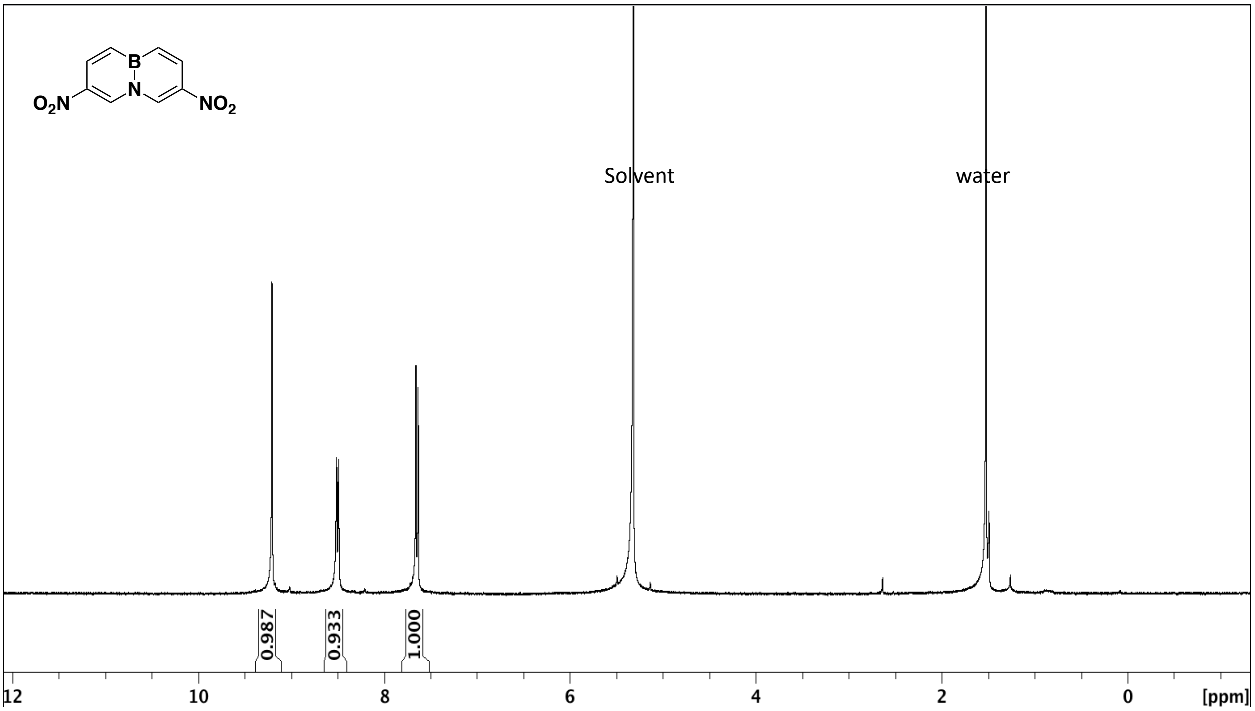
O2N
B
N
NO2
Solvent
water
0.987
0.933
1.000
12
10
8
6
4
2
0
[ppm]

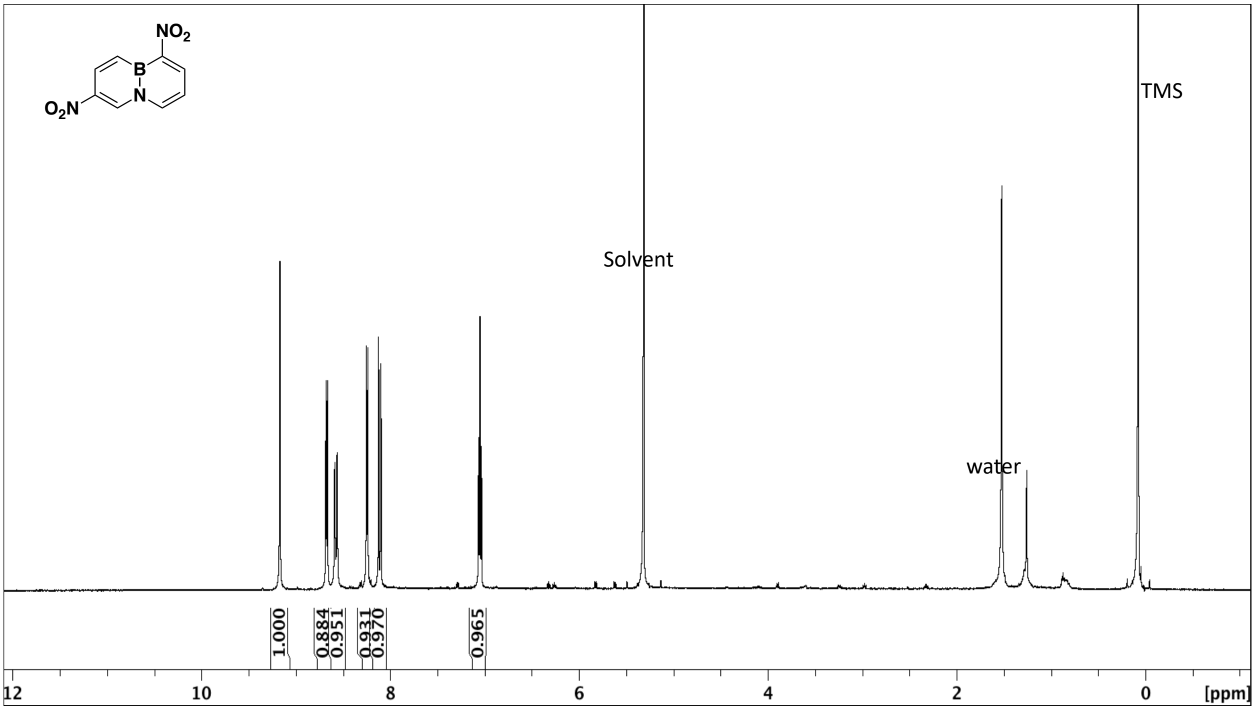
NO2
B
N
O2N
Solvent
TMS
water
1.000
0.884
0.951
0.931
0.970
0.965
12
10
8
6
4
2
0
[ppm]

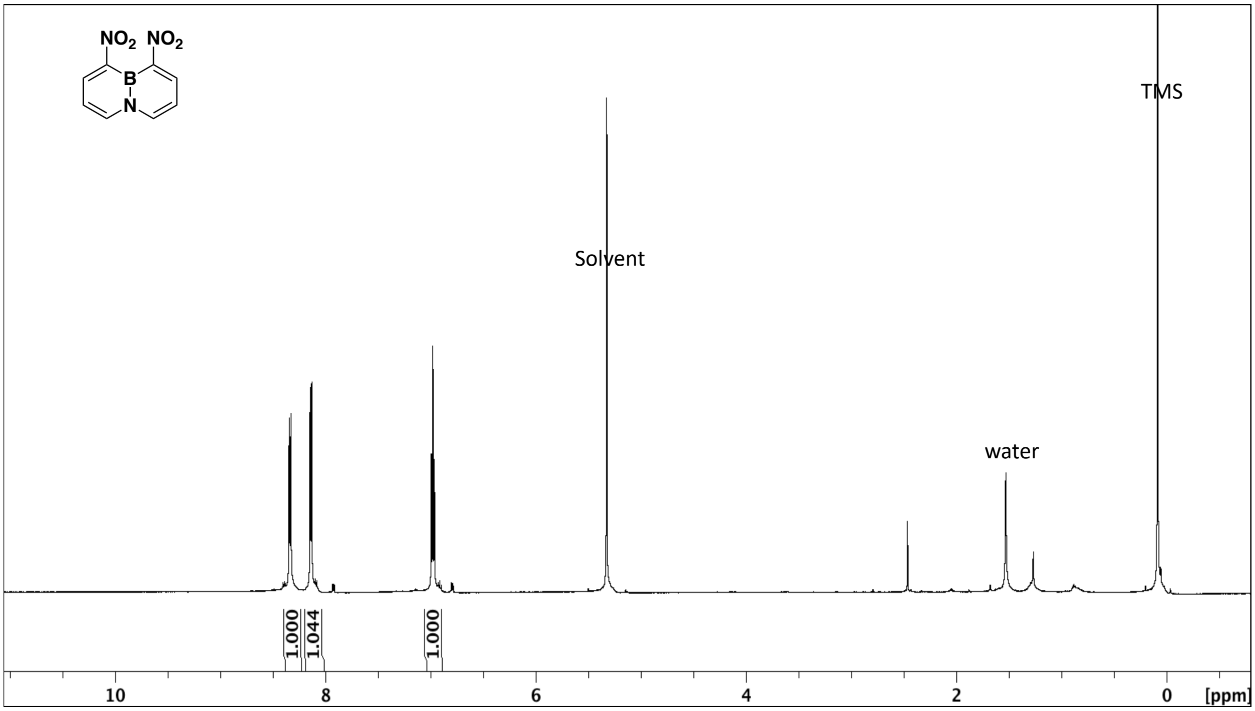

2. *C-13 NMR spectra of compound **3**, **4**, **5**, **6**, and **7**.*

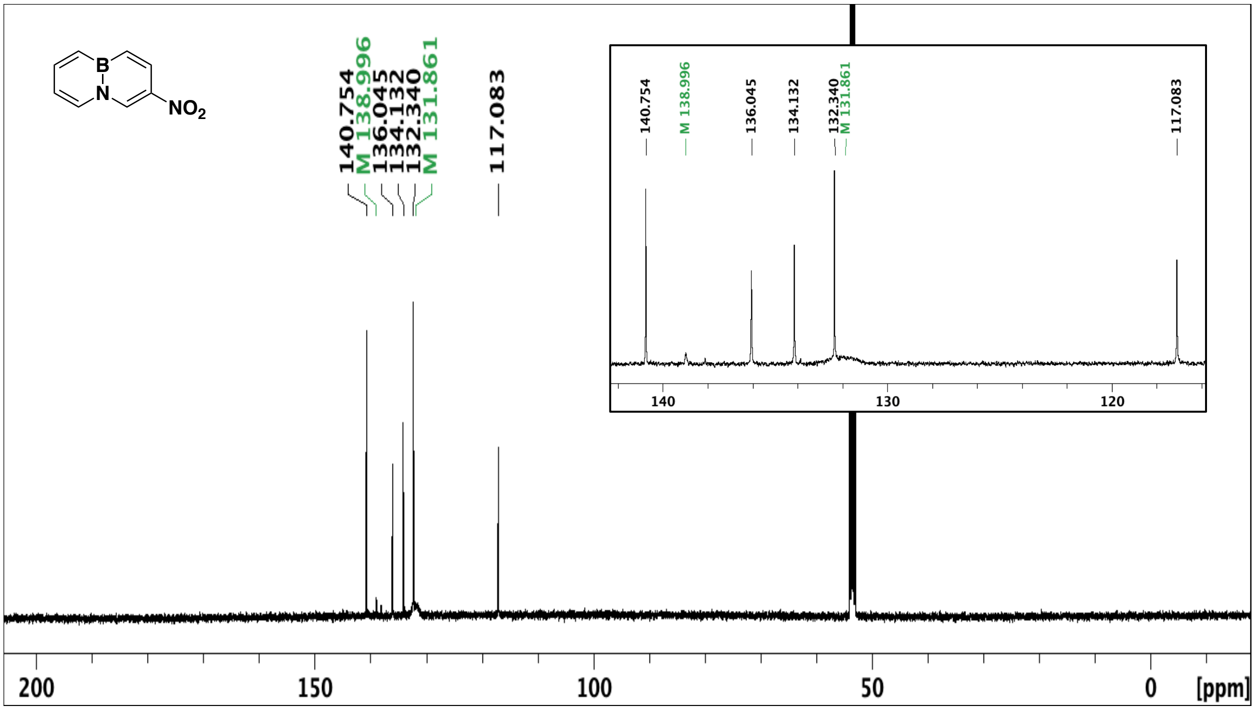

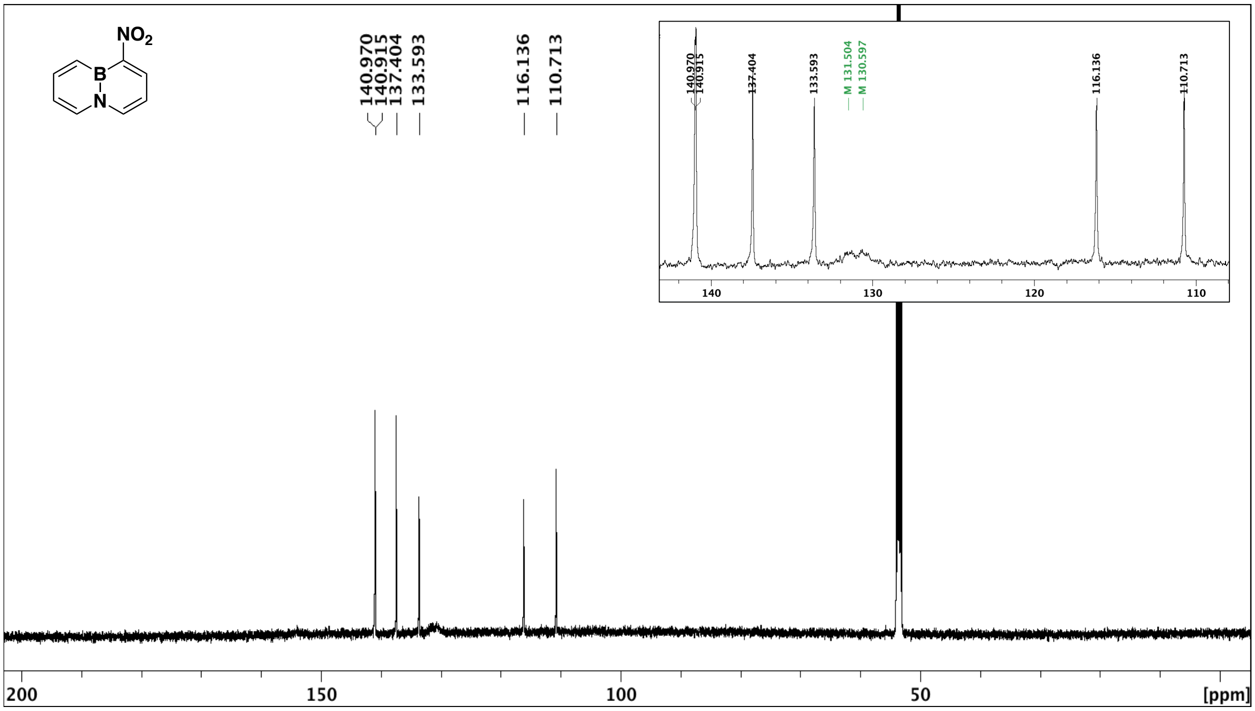
NO2
B
N
140.970
140.915
137.404
133.593
116.136
110.713
M 131.504
M 130.597
140
130
120
110
200
150
100
50
[ppm]

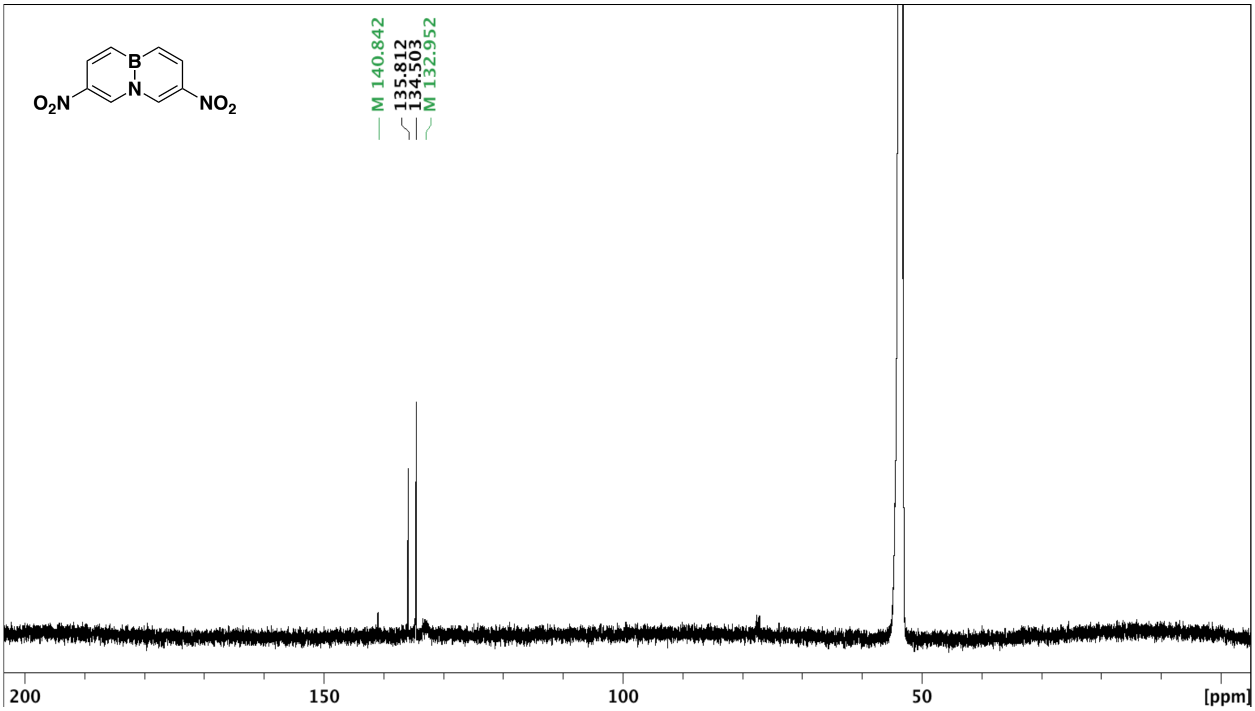
O2N
B
N
NO2
M 140.842
135.812
134.503
M 132.952
200
150
100
50
[ppm]

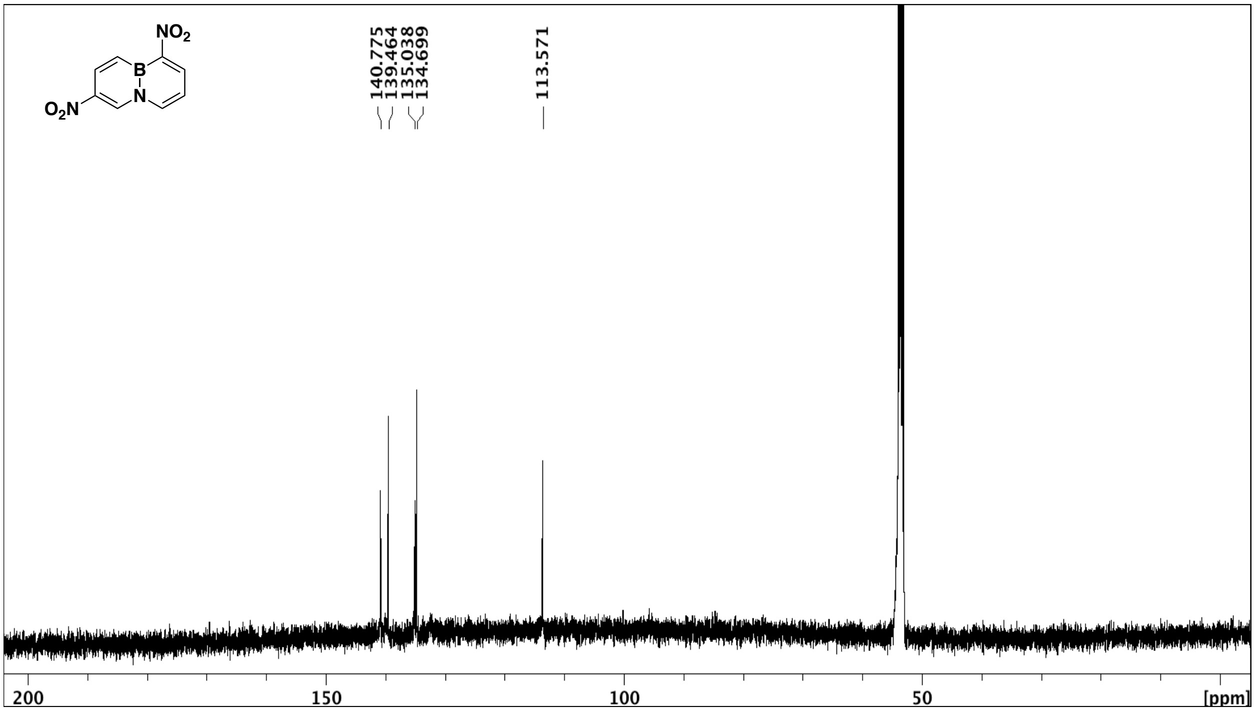
NO2
B
N
O2N
140.775
139.464
135.038
134.699
113.571
200
150
100
50
[ppm]

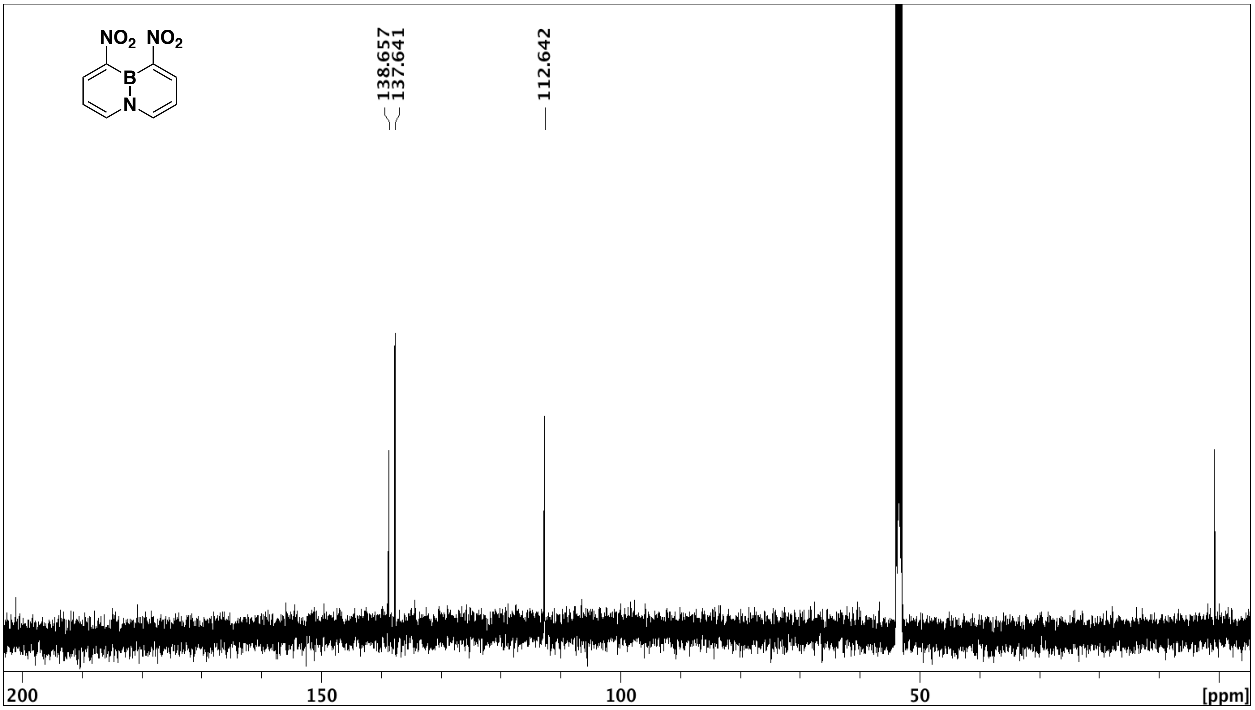
NO2
NO2
B
N
138.657
137.641
112.642
200
150
100
50
[ppm]

3. *B-11 NMR spectra of compound **3**, **4**, **5**, **6**, and 7.*

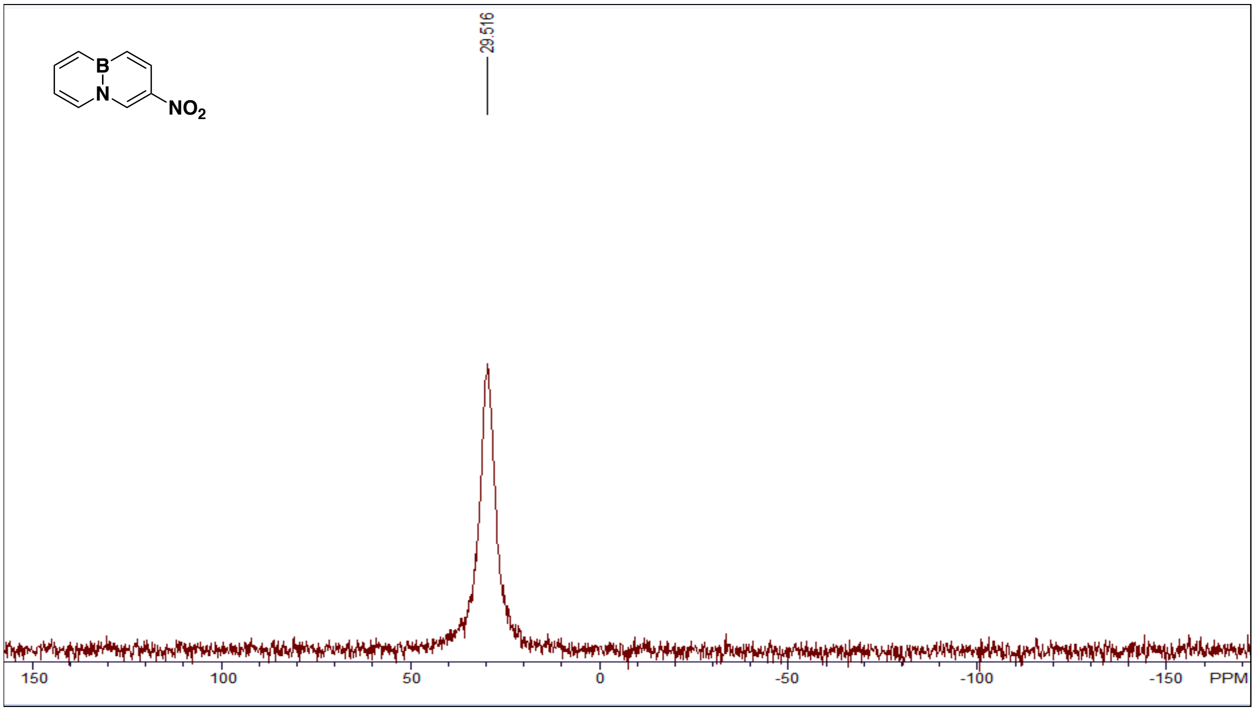

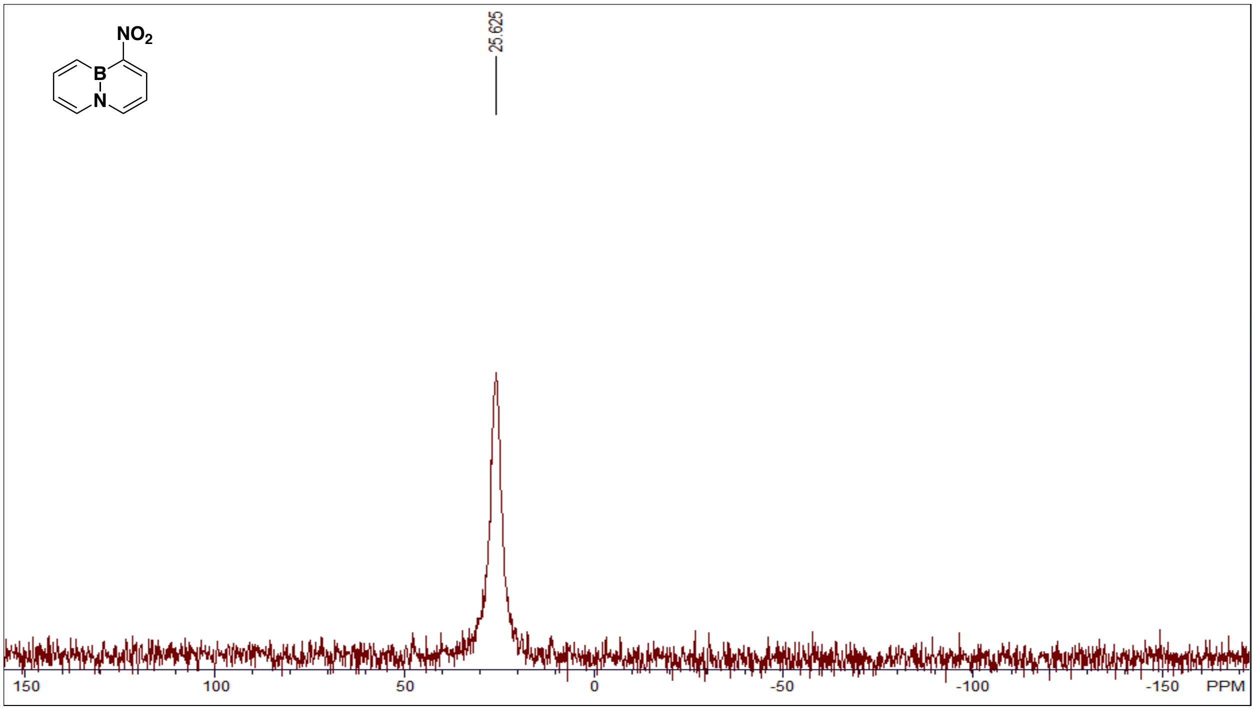

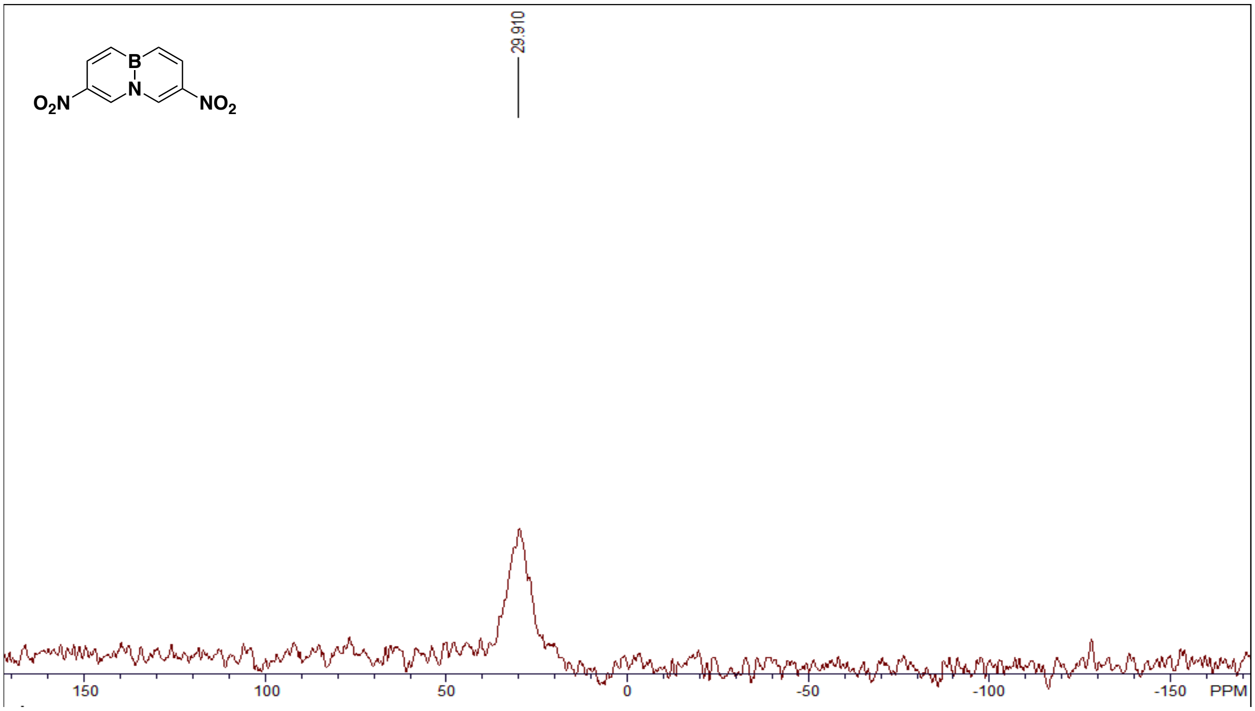
O2N
NO2
B
N
29.910
150
100
50
0
-50
-100
-150
PPM

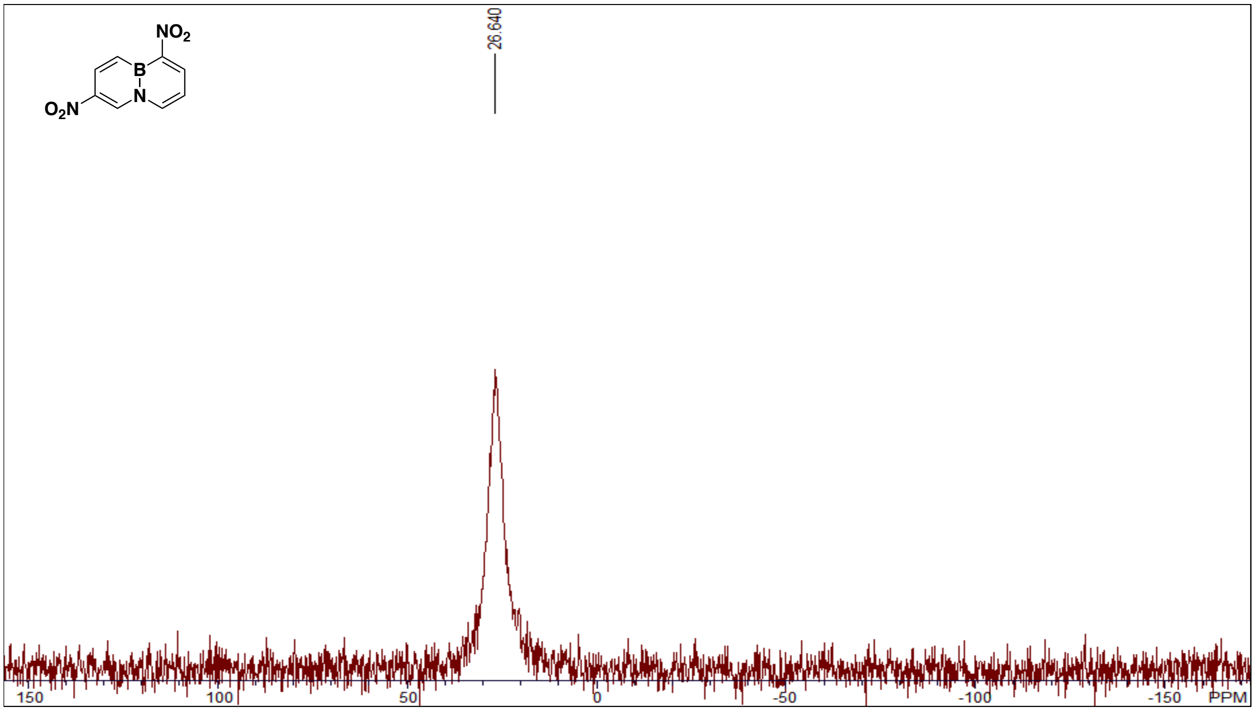
NO2
O2N
B
N
26.640
150
100
50
0
-50
-100
-150
PPM

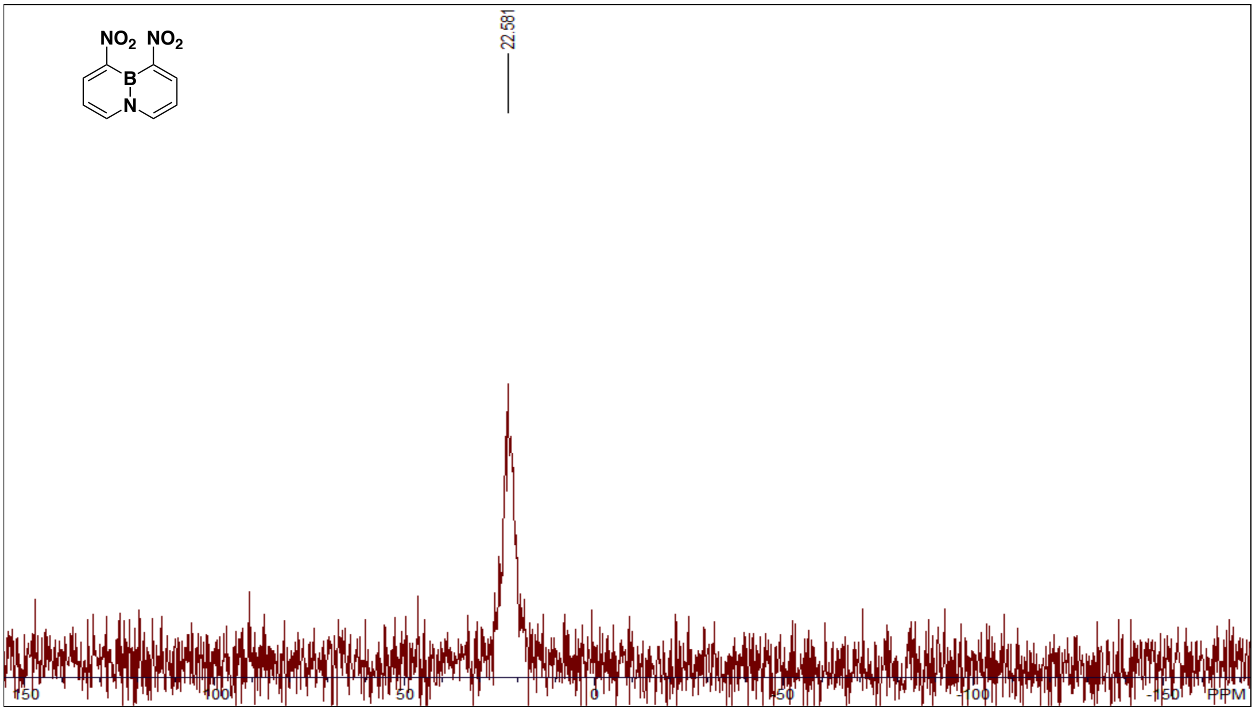

4. *Infrared spectrum of compound **3**, **4**, **5**, **6**, and **7**.*

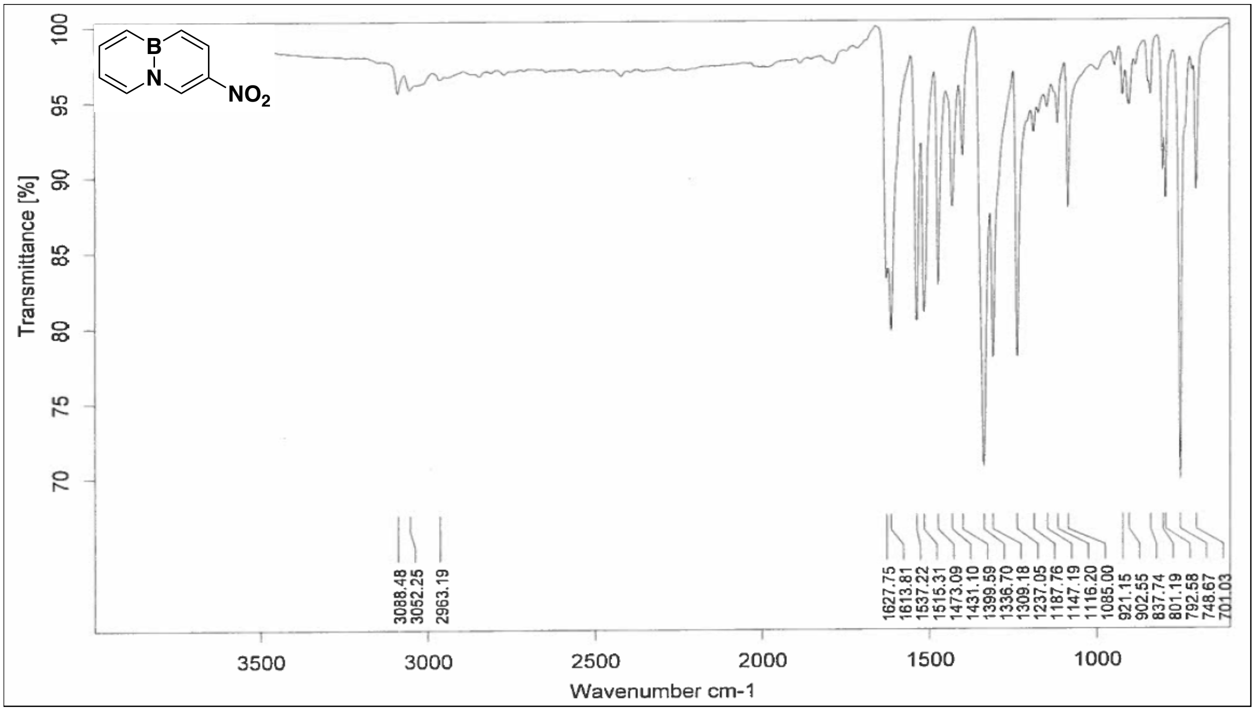

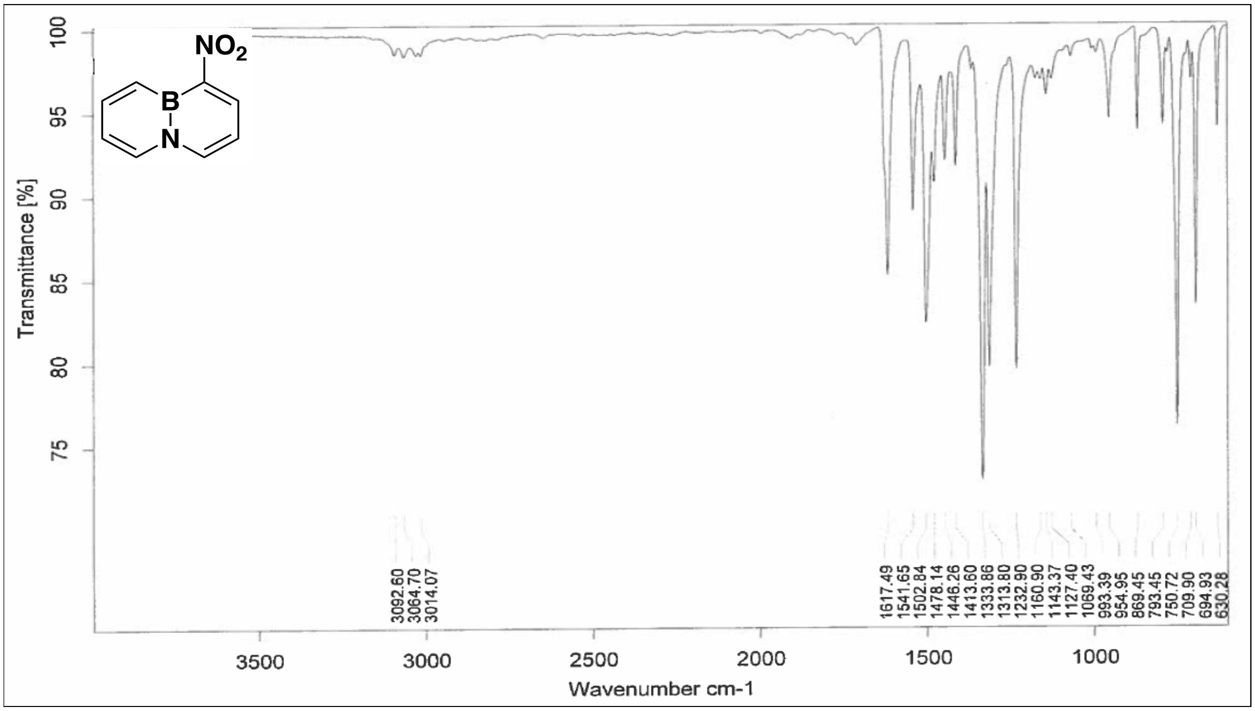
NO2
B
N
Transmittance [%]
Wavenumber cm-1
3092.60
3064.70
3014.07
1617.49
1541.65
1502.84
1478.14
1446.26
1413.60
1333.86
1313.80
1232.90
1160.90
1143.37
1127.40
1069.43
993.39
954.95
869.45
793.45
750.72
709.90
694.93
630.28

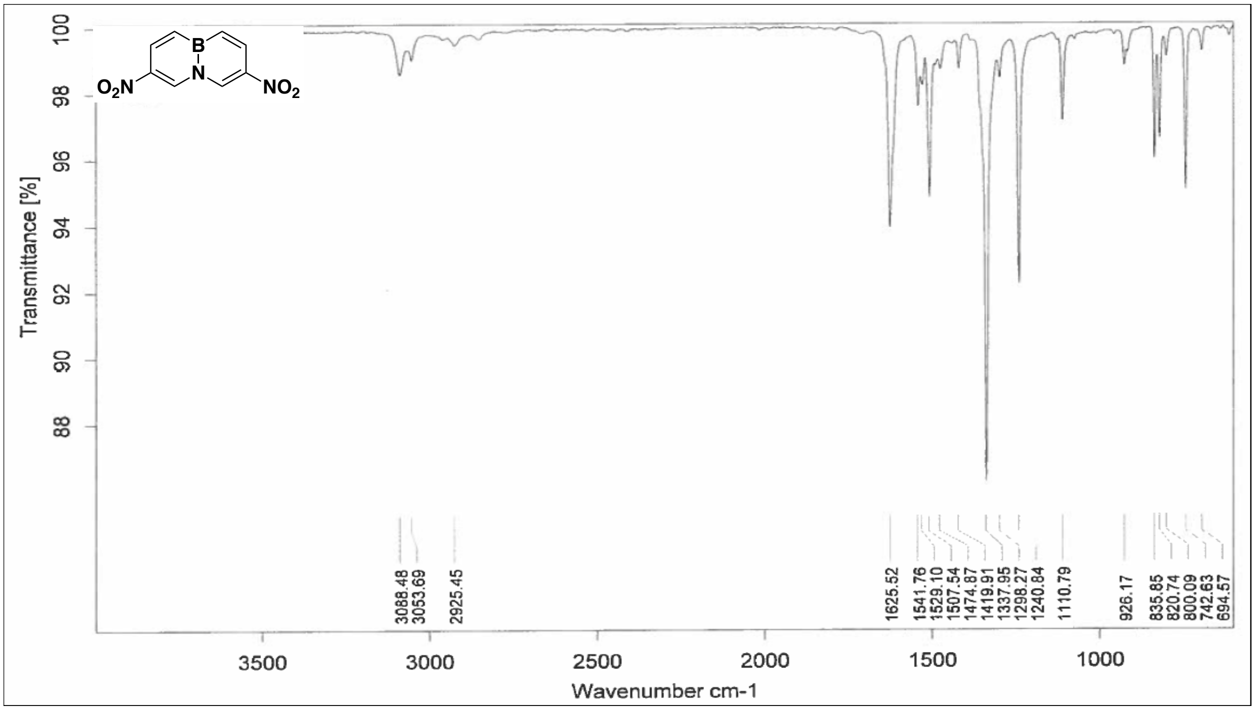
O2N
B
N
NO2
Transmittance [%]
Wavenumber cm-1
3088.48
3053.69
2925.45
1625.52
1541.76
1529.10
1507.54
1474.87
1419.91
1337.95
1298.27
1240.84
1110.79
926.17
835.85
820.74
800.09
742.63
694.57

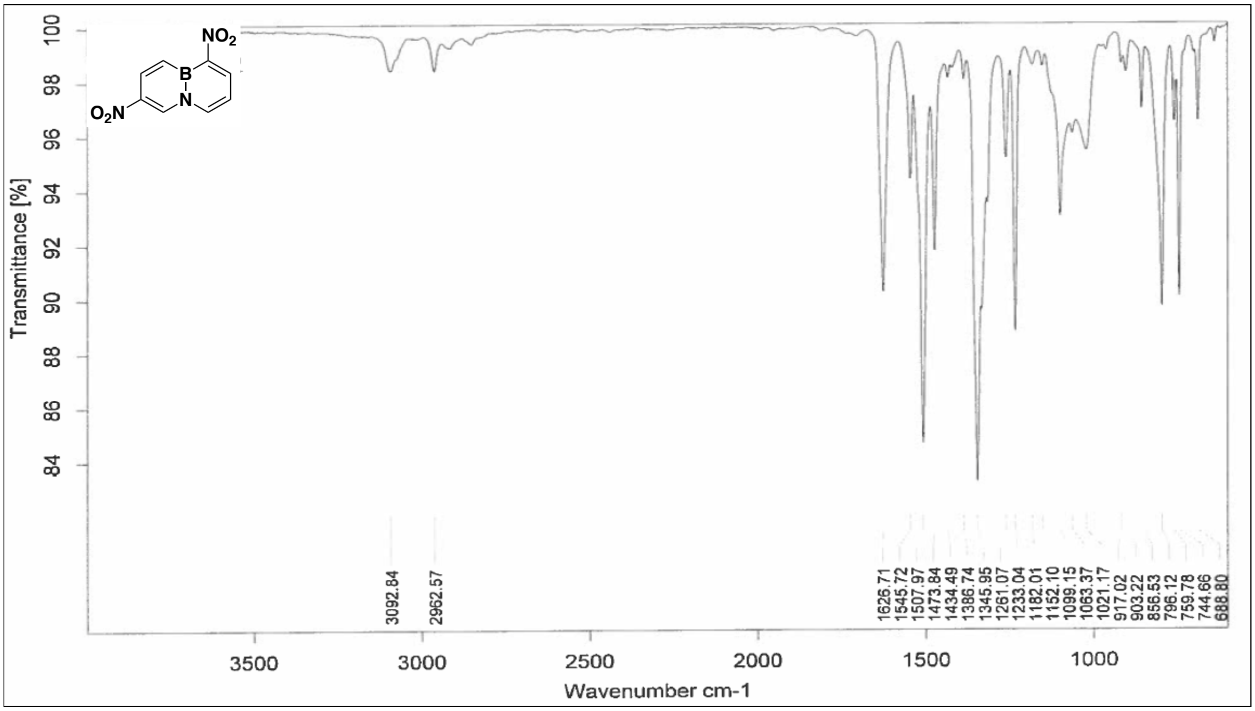
NO2
O2N
B
N
Transmittance [%]
100
98
96
94
92
90
88
86
84
3092.84
2962.57
1626.71
1545.72
1507.97
1473.84
1434.49
1386.74
1345.95
1261.07
1233.04
1182.01
1152.10
1099.15
1063.37
1021.17
917.02
903.22
856.53
796.12
759.78
744.66
688.80
3500
3000
2500
2000
1500
1000
Wavenumber cm-1

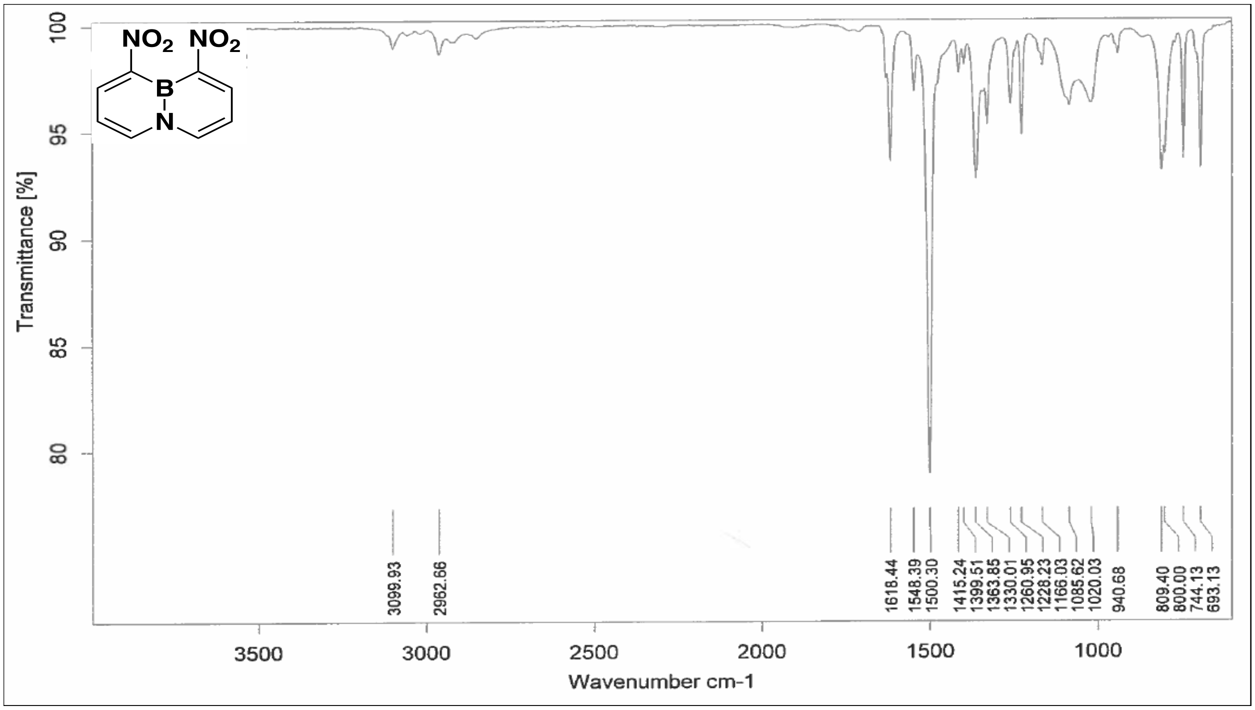
NO2
NO2
B
N
Transmittance [%]
100
95
90
85
80
3099.93
2962.66
1618.44
1548.39
1500.30
1415.24
1399.51
1363.85
1330.01
1260.95
1228.23
1166.03
1085.62
1020.03
940.68
809.40
800.00
744.13
693.13
3500
3000
2500
2000
1500
1000
Wavenumber cm-1

5. *X-ray crystallographic analysis data for **1-nitro-BNN, 1,6-dinitro-BNN and 1,8-dinitro-BNN***
6.
Table S1, Crystal data and structure refinement for 1-nitro-BNN **1-nitro-BNN**.

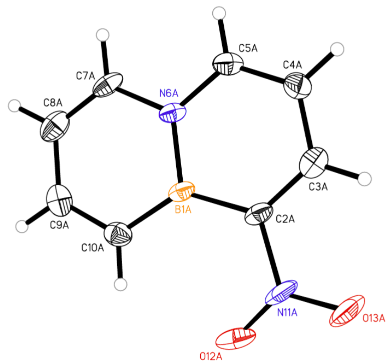

Empirical formula	$C_8H_7BN_2O_2$	
Formula weight	173.97	
Temperature	150(2) K	
Wavelength	0.71073 Å	
Crystal system	Orthorhombic	
Space group	$Pca2_1$	
Unit cell dimensions	a = 16.722(2) Å	α= 90°.
	b = 3.7994(5) Å	β= 90°.
	c = 25.070(3) Å	γ = 90°.
Volume	1592.8(4) Å^3	
Z	8	
Density (-123ºC)	1.451 Mg/m^3	
Density (20ºC)	1.406 Mg/m^3	
Absorption coefficient	0.104 mm^{-1}	
F(000)	720	
Crystal size	0.62 x 0.14 x 0.08 mm^3	
Theta range for data collection	2.44 to 26.52°.	
Index ranges	-20<=h<=20, -4<=k<=4, -31<=l<=27	
Reflections collected	12754	
Independent reflections	3068 [R_{int} = 0.0652]	
Completeness to theta = 26.52°	99.1 %	
Absorption correction	Semi-empirical from equivalents	
Max. and min. transmission	0.9917 and 0.9383	
Refinement method	Full-matrix least-squares on F^2	
Data / restraints / parameters	3068 / 1 / 235	
Goodness-of-fit on F^2	1.101	
Final R indices [I>2sigma(I)]	R_1 = 0.0546, wR_2 = 0.1352	
R indices (all data)	R_1 = 0.0628, wR_2 = 0.1416	
Largest diff. peak and hole	0.363 and -0.275 e.Å^{-3}	

Table S2. Bond lengths [Å] and angles [°] for 1-nitro-BNN **1-nitro-BNN**.

B(1A)-N(6A)	1.472(3)	B(1A)-C(10A)	1.510(5)
B(1A)-C(2A)	1.525(4)	C(2A)-C(3A)	1.358(5)
C(2A)-N(11A)	1.474(3)	C(3A)-C(4A)	1.415(4)
C(3A)-H(3A)	0.9500	C(4A)-C(5A)	1.359(4)
C(4A)-H(4A)	0.9500	C(5A)-N(6A)	1.382(4)
C(5A)-H(5A)	0.9500	N(6A)-C(7A)	1.395(3)
C(7A)-C(8A)	1.343(5)	C(7A)-H(7A)	0.9500
C(8A)-C(9A)	1.427(4)	C(8A)-H(8A)	0.9500
C(9A)-C(10A)	1.364(5)	C(9A)-H(9A)	0.9500
C(10A)-H(10A)	0.9500	N(11A)-O(12A)	1.226(4)
N(11A)-O(13A)	1.231(4)	B(1B)-N(6B)	1.473(4)
B(1B)-C(10B)	1.521(5)	B(1B)-C(2B)	1.531(4)
C(2B)-C(3B)	1.372(5)	C(2B)-N(11B)	1.453(3)
C(3B)-C(4B)	1.410(4)	C(3B)-H(3B)	0.9500
C(4B)-C(5B)	1.351(5)	C(4B)-H(4B)	0.9500
C(5B)-N(6B)	1.372(4)	C(5B)-H(5B)	0.9500
N(6B)-C(7B)	1.392(3)	C(7B)-C(8B)	1.346(5)
C(7B)-H(7B)	0.9500	C(8B)-C(9B)	1.420(5)
C(8B)-H(8B)	0.9500	C(9B)-C(10B)	1.347(5)
C(9B)-H(9B)	0.9500	C(10B)-H(10B)	0.9500
N(11B)-O(13B)	1.235(4)	N(11B)-O(12B)	1.235(4)
N(6A)-B(1A)-C(10A)	115.5(2)	N(6A)-B(1A)-C(2A)	112.0(2)
C(10A)-B(1A)-C(2A)	132.5(2)	C(3A)-C(2A)-N(11A)	115.8(2)
C(3A)-C(2A)-B(1A)	122.4(2)	N(11A)-C(2A)-B(1A)	121.8(2)
C(2A)-C(3A)-C(4A)	120.4(3)	C(2A)-C(3A)-H(3A)	119.8
C(4A)-C(3A)-H(3A)	119.8	C(5A)-C(4A)-C(3A)	120.9(3)
C(5A)-C(4A)-H(4A)	119.6	C(3A)-C(4A)-H(4A)	119.6
C(4A)-C(5A)-N(6A)	121.8(2)	C(4A)-C(5A)-H(5A)	119.1
N(6A)-C(5A)-H(5A)	119.1	C(5A)-N(6A)-C(7A)	117.1(2)
C(5A)-N(6A)-B(1A)	122.5(2)	C(7A)-N(6A)-B(1A)	120.3(2)
C(8A)-C(7A)-N(6A)	121.6(2)	C(8A)-C(7A)-H(7A)	119.2
N(6A)-C(7A)-H(7A)	119.2	C(7A)-C(8A)-C(9A)	121.9(3)
C(7A)-C(8A)-H(8A)	119.0	C(9A)-C(8A)-H(8A)	119.0
C(10A)-C(9A)-C(8A)	120.6(3)	C(10A)-C(9A)-H(9A)	119.7
C(8A)-C(9A)-H(9A)	119.7	C(9A)-C(10A)-B(1A)	120.0(3)
C(9A)-C(10A)-H(10A)	120.0	B(1A)-C(10A)-H(10A)	120.0
O(12A)-N(11A)-O(13A)	122.6(3)	O(12A)-N(11A)-C(2A)	118.0(2)
O(13A)-N(11A)-C(2A)	119.4(3)	N(6B)-B(1B)-C(10B)	115.6(3)
N(6B)-B(1B)-C(2B)	112.3(3)	C(10B)-B(1B)-C(2B)	132.1(3)

C(3B)-C(2B)-N(11B)	116.0(3)	C(3B)-C(2B)-B(1B)	121.9(2)
N(11B)-C(2B)-B(1B)	122.1(3)	C(2B)-C(3B)-C(4B)	119.7(3)
C(2B)-C(3B)-H(3B)	120.2	C(4B)-C(3B)-H(3B)	120.2

Table S2. Continued

C(5B)-C(4B)-C(3B)	121.5(3)	C(5B)-C(4B)-H(4B)	119.2
C(3B)-C(4B)-H(4B)	119.2	C(4B)-C(5B)-N(6B)	122.3(3)
C(4B)-C(5B)-H(5B)	118.8	N(6B)-C(5B)-H(5B)	118.8
C(5B)-N(6B)-C(7B)	117.3(2)	C(5B)-N(6B)-B(1B)	122.2(2)
C(7B)-N(6B)-B(1B)	120.5(3)	C(8B)-C(7B)-N(6B)	121.2(3)
C(8B)-C(7B)-H(7B)	119.4	N(6B)-C(7B)-H(7B)	119.4
C(7B)-C(8B)-C(9B)	121.7(3)	C(7B)-C(8B)-H(8B)	119.2
C(9B)-C(8B)-H(8B)	119.2	C(10B)-C(9B)-C(8B)	122.1(4)
C(10B)-C(9B)-H(9B)	119.0	C(8B)-C(9B)-H(9B)	119.0
C(9B)-C(10B)-B(1B)	118.9(3)	C(9B)-C(10B)-H(10B)	120.5
B(1B)-C(10B)-H(10B)	120.5	O(13B)-N(11B)-O(12B)	121.6(3)
O(13B)-N(11B)-C(2B)	120.1(3)	O(12B)-N(11B)-C(2B)	118.3(3)

Table S3. Crystal data and structure refinement for 1,6-dinitro-BNN **1,6-dinitro-BNN**.

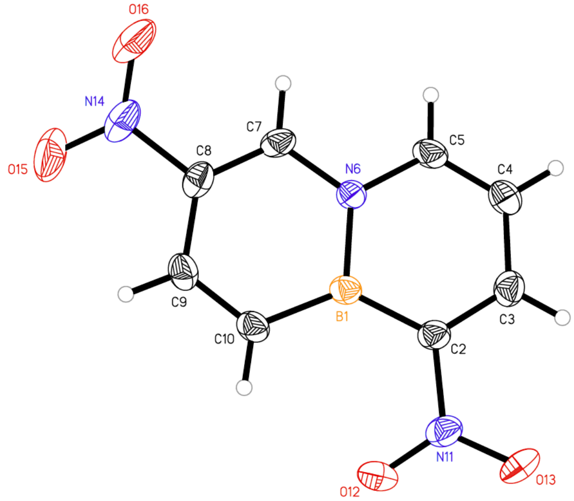

Empirical formula	$C_8H_6BN_3O_4$	
Formula weight	218.97	
Temperature	293(2) K	
Wavelength	0.71073 Å	
Crystal system	Orthorhombic	
Space group	$Pna2_1$	
Unit cell dimensions	a = 15.0780(17) Å	α= 90°.
	b = 16.2287(18) Å	β= 90°.
	c = 3.7551(5) Å	γ = 90°.
Volume	918.86(19) $Å^3$	
Z	4	
Density (calculated)	1.583 Mg/m^3	
Absorption coefficient	0.127 mm^{-1}	
F(000)	448	
Crystal size	0.41 x 0.05 x 0.04 mm^3	
Theta range for data collection	1.84 to 26.51°.	
Index ranges	-18<=h<=18, -14<=k<=20, -4<=l<=4	
Reflections collected	6470	
Independent reflections	1879 [R_{int} = 0.0505]	
Completeness to theta = 26.51°	98.6 %	
Absorption correction	Semi-empirical from equivalents	
Max. and min. transmission	0.9949 and 0.9499	
Refinement method	Full-matrix least-squares on F^2	
Data / restraints / parameters	1879 / 1 / 145	
Goodness-of-fit on F^2	1.051	
Final R indices [I>2sigma(I)]	R_1 = 0.0467, wR_2 = 0.1133	
R indices (all data)	R_1 = 0.0632, wR_2 = 0.1241	

Largest diff. peak and hole 0.237 and -0.195 e.Å^{-3}

Table S4. Bond lengths [Å] and angles [°] for 1,6-dinitro-BNN **1,6-dinitro-BNN**.

B(1)-N(6)	1.469(3)	B(1)-C(2)	1.517(4)
B(1)-C(10)	1.520(4)	C(2)-C(3)	1.368(4)
C(2)-N(11)	1.460(3)	C(3)-C(4)	1.412(4)
C(3)-H(3)	0.9300	C(4)-C(5)	1.344(4)
C(4)-H(4)	0.9300	C(5)-N(6)	1.395(3)
C(5)-H(5)	0.9300	N(6)-C(7)	1.381(3)
C(7)-C(8)	1.353(4)	C(7)-H(7)	0.9300
C(8)-C(9)	1.423(4)	C(8)-N(14)	1.468(3)
C(9)-C(10)	1.350(4)	C(9)-H(9)	0.9300
C(10)-H(10)	0.9300	N(11)-O(12)	1.228(3)
N(11)-O(13)	1.234(3)	N(14)-O(15)	1.225(3)
N(14)-O(16)	1.225(3)		
N(6)-B(1)-C(2)	113.2(2)	N(6)-B(1)-C(10)	115.4(2)
C(2)-B(1)-C(10)	131.3(2)	C(3)-C(2)-N(11)	116.3(2)
C(3)-C(2)-B(1)	121.6(2)	N(11)-C(2)-B(1)	122.1(2)
C(2)-C(3)-C(4)	120.0(2)	C(2)-C(3)-H(3)	120.0
C(4)-C(3)-H(3)	120.0	C(5)-C(4)-C(3)	122.0(2)
C(5)-C(4)-H(4)	119.0	C(3)-C(4)-H(4)	119.0
C(4)-C(5)-N(6)	121.5(2)	C(4)-C(5)-H(5)	119.2
N(6)-C(5)-H(5)	119.2	C(7)-N(6)-C(5)	117.2(2)
C(7)-N(6)-B(1)	121.2(2)	C(5)-N(6)-B(1)	121.6(2)
C(8)-C(7)-N(6)	119.5(2)	C(8)-C(7)-H(7)	120.2
N(6)-C(7)-H(7)	120.2	C(7)-C(8)-C(9)	124.4(2)
C(7)-C(8)-N(14)	116.9(2)	C(9)-C(8)-N(14)	118.7(2)
C(10)-C(9)-C(8)	119.1(2)	C(10)-C(9)-H(9)	120.5
C(8)-C(9)-H(9)	120.5	C(9)-C(10)-B(1)	120.3(2)
C(9)-C(10)-H(10)	119.9	B(1)-C(10)-H(10)	119.9
O(12)-N(11)-O(13)	121.9(2)	O(12)-N(11)-C(2)	118.5(2)
O(13)-N(11)-C(2)	119.6(2)	O(15)-N(14)-O(16)	123.9(2)
O(15)-N(14)-C(8)	117.4(2)	O(16)-N(14)-C(8)	118.7(2)

Table S5. Crystal data and structure refinement for 1,8-dinitro-BNN **1,8-dinitro-BNN**

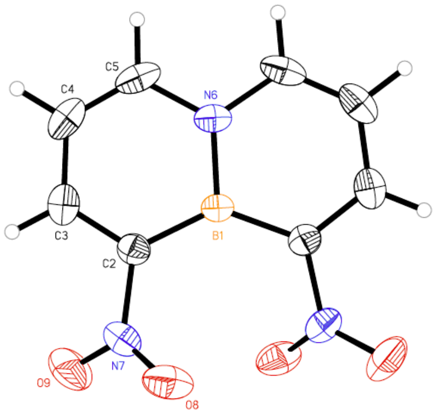

Empirical formula	$C_8H_6BN_3O_4$	
Formula weight	218.97	
Temperature	293(2) K	
Wavelength	0.71073 Å	
Crystal system	Monoclinic	
Space group	C2/c	
Unit cell dimensions	a = 14.541(5) Å	α= 90°.
	b = 8.153(2) Å	β= 117.709(15)°.
	c = 8.923(2) Å	γ = 90°.
Volume	936.6(5) Å^3	
Z	4	
Density (calculated)	1.553 Mg/m^3	
Absorption coefficient	0.124 mm^{-1}	
F(000)	448	
Crystal size	0.80 x 0.18 x 0.08 mm^3	
Theta range for data collection	2.96 to 26.13°.	
Index ranges	-17<=h<=17, -10<=k<=9, -10<=l<=10	
Reflections collected	4108	
Independent reflections	917 [R_{int} = 0.0184]	
Completeness to theta = 26.13°	98.4 %	
Absorption correction	Semi-empirical from equivalents	
Max. and min. transmission	0.9901 and 0.9071	
Refinement method	Full-matrix least-squares on F^2	
Data / restraints / parameters	917 / 0 / 74	
Goodness-of-fit on F^2	1.108	
Final R indices [I>2sigma(I)]	R_1 = 0.0448, wR_2 = 0.1222	
R indices (all data)	R_1 = 0.0514, wR_2 = 0.1283	
Largest diff. peak and hole	0.189 and -0.206 e.Å^{-3}	

Table S6. Bond lengths [Å] and angles [°] for 1,8-dinitro-BNN **1,8-dinitro-BNN**

B(1)-N(6)	1.460(3)
B(1)-C(2)	1.5195(19)
B(1)-C(2)#1	1.5195(19)
C(2)-C(3)	1.356(3)
C(2)-N(7)	1.457(2)
C(3)-C(4)	1.396(3)
C(3)-H(3)	0.9500
C(4)-C(5)	1.337(3)
C(4)-H(4)	0.9500
C(5)-N(6)	1.387(2)
C(5)-H(5)	0.9500
N(6)-C(5)#1	1.387(2)
N(7)-O(9)	1.216(2)
N(7)-O(8)	1.226(2)
N(6)-B(1)-C(2)	113.14(11)
N(6)-B(1)-C(2)#1	113.14(11)
C(2)-B(1)-C(2)#1	133.7(2)
C(3)-C(2)-N(7)	115.39(16)
C(3)-C(2)-B(1)	121.47(18)
N(7)-C(2)-B(1)	123.04(16)
C(2)-C(3)-C(4)	120.26(18)
C(2)-C(3)-H(3)	119.9
C(4)-C(3)-H(3)	119.9
C(5)-C(4)-C(3)	121.79(18)
C(5)-C(4)-H(4)	119.1
C(3)-C(4)-H(4)	119.1
C(4)-C(5)-N(6)	122.04(18)
C(4)-C(5)-H(5)	119.0
N(6)-C(5)-H(5)	119.0
C(5)-N(6)-C(5)#1	117.6(2)
C(5)-N(6)-B(1)	121.20(12)
C(5)#1-N(6)-B(1)	121.20(12)
O(9)-N(7)-O(8)	122.79(18)
O(9)-N(7)-C(2)	119.85(17)
O(8)-N(7)-C(2)	117.30(15)
